# Exposed seronegative: Cellular immune responses to SARS-CoV-2 in the absence of seroconversion

**DOI:** 10.3389/fimmu.2023.1092910

**Published:** 2023-01-26

**Authors:** Cecilia Jay, Jeremy Ratcliff, Lance Turtle, Philip Goulder, Paul Klenerman

**Affiliations:** ^1^ Peter Medawar Building for Pathogen Research, Nuffield Department of Medicine, University of Oxford, Oxford, United Kingdom; ^2^ National Institute for Health and Care Research Health Protection Research Unit in Emerging and Zoonotic Infections, University of Liverpool, Liverpool, United Kingdom; ^3^ Peter Medawar Building for Pathogen Research, Department of Paediatrics, University of Oxford, Oxford, United Kingdom

**Keywords:** SARS-CoV-2, seronegative, T-cells, exposed, hepatitis C

## Abstract

The factors determining whether infection will occur following exposure to SARS-CoV-2 remain elusive. Certain SARS-CoV-2-exposed individuals mount a specific T-cell response but fail to seroconvert, representing a population that may provide further clarity on the nature of infection susceptibility and correlates of protection against SARS-CoV-2. Exposed seronegative individuals have been reported in patients exposed to the blood-borne pathogens Human Immunodeficiency virus and Hepatitis C virus and the sexually transmitted viruses Hepatitis B virus and Herpes Simplex virus. By comparing the quality of seronegative T-cell responses to SARS-CoV-2 with seronegative cellular immunity to these highly divergent viruses, common patterns emerge that offer insights on the role of cellular immunity against infection. For both SARS-CoV-2 and Hepatitis C, T-cell responses in exposed seronegatives are consistently higher than in unexposed individuals, but lower than in infected, seropositive patients. Durability of T-cell responses to Hepatitis C is dependent upon repeated exposure to antigen – single exposures do not generate long-lived memory T-cells. Finally, exposure to SARS-CoV-2 induces varying degrees of immune activation, suggesting that exposed seronegative individuals represent points on a spectrum rather than a discrete group. Together, these findings paint a complex landscape of the nature of infection but provide clues as to what may be protective early on in SARS-CoV-2 disease course. Further research on this phenomenon, particularly through cohort studies, is warranted.

## Introduction

Exposure to viral pathogens does not guarantee infection. The clearest examples of this phenomenon are in the failure of test subjects in human challenge studies to consistently become infected (1–3). Variation in host susceptibility has been linked to host genetics, inoculum viral load, and prior exposure to related pathogens (2–6). Among those individuals who are exposed but fail to become infected, a small but well-documented population generate pathogen-specific T-cell responses in the absence of viraemia or antibodies (7–9). The earliest reports of this phenomenon occurred in the late 1980s concerning apparent Human Immunodeficiency virus (HIV) resistance in at-risk individuals (10–14). These patients, despite exposure to HIV and measurable cellular immunity, failed to develop an antibody response and were therefore classified as “exposed seronegative” (ESN). In the 30 years since initial reports in HIV, the phenomenon has been appreciated to occur following exposure to Hepatitis C virus (HCV), Hepatitis B virus (HBV), and Herpes Simplex virus 2 (HSV-2) (8, 15–18), and recently Severe Acute Respiratory Syndrome coronavirus 2 (SARS-CoV-2), a pathogen to which most of the global population has been exposed (7, 19, 20). SARS-CoV-2, a member of the *Coronaviridae* family, is the causative agent of coronavirus disease 2019 (COVID-19). Canonical immunity to SARS-CoV-2 is well characterised despite its recent emergence: a delay in innate immune activation resulting from viral evasion of interferon (IFN) responses enables infection to occur (21). Both humoral and cellular mechanisms are essential for viral control; weak or delayed adaptive responses can lead to severe or fatal COVID-19, with immunopathology and cytokine hyperactivation characteristic of end-stage disease (21).

The causes and consequences of the ESN phenomenon following exposure to SARS-CoV-2, as well as other viruses, are the focus of this review. We outline the circumstances in which seronegative cellular immunity occurs and examine the quality of the T-cell response. In addition, we address the role of viral exposure on response durability, and whether this offers protection against infection. Finally, potential mechanisms are discussed, as well as gaps in current knowledge that future research must fill.

## Definitions and terminology

For this review, an ESN is defined as an individual who mounts a cellular immune response following viral exposure without generating detectable virus-specific antibody. Instances where infection has been prevented by the innate immune system alone, as reported elsewhere (22, 23), are out of scope for this review due to not inducing a T-cell response. In the literature, the terminology for ESNs is largely consistent within but not between viruses and include exposed seronegative(s), exposed uninfected, infected seronegative, immune seronegative, and highly exposed persistently seronegative. These terms will be collectively referred to as ESN.

## At-risk demographics

The dynamics of exposure, such as inoculum viral load, exposure frequency, and exposure duration, may influence cellular responses in ESNs. To make robust comparisons between SARS-CoV-2 and other viruses, we examine cellular immunity in two demographics at high risk of exposure – close contacts of seropositive individuals, and healthcare workers (HCWs). This enables the analysis of common and differing patterns of immunity to unrelated viruses through similar modes of exposure.

## Close and household contacts

Close contacts of SARS-CoV-2-infected individuals represent a population exposed to SARS-CoV-2 over short time periods. Wang et al. (2021) assessed cellular immunity in 90 seropositive individuals and 69 seronegative contacts who had been within 1.5m of a patient for over one hour or in the same household for over 24 hours (20). The authors observed higher CD8^+^ and CD4^+^ T-cell activation in both cohorts compared to unexposed controls, as measured by IFN*γ* production following stimulation with Spike (S), Membrane (M), Nucleocapsid (NP), and Envelope peptides. Gallais et al. (2021) also identified T-cell responses in close contacts of SARS-CoV-2-infected family members (24).

Family members of HCV-infected individuals face viral exposure through sexual and *in-utero* transmission. Kamal et al. (2004) identified 14 seronegative sexual partners of HCV-infected individuals who generated IFN*γ* responses to HCV, although at lower magnitudes than seropositive resolvers (25). HCV is a positive-sense RNA virus in the *Flaviviridae* family (26). Canonically, adaptive immune responses appear one to two months after infection (27), and both CD4^+^ and CD8^+^ T-cells are associated with control (26). In individuals exposed to HCV *via* a family member, Scognamiglio et al. (1999) identified CD8^+^ T-cells targeting both structural and non-structural proteins (NSPs) (16). There was no correlation between magnitude of response and mode of exposure, suggesting that similar cellular responses are generated following HCV exposure through different routes.

HCWs represent a population where SARS-CoV-2 exposure in clinical settings enables the study of ESNs. da Silva Antunes et al. (2021) recruited 26 PCR+ HCWs, 32 seronegative HCWs at high risk of exposure (treated as ESNs here), and 33 community controls (28). ESNs demonstrated higher T-cell responses than unexposed individuals. Notably, the T-cell activation markers HLA-DR and CD38 were upregulated in PCR+ HCWs, but not in ESNs, leading authors to conclude that cellular responses in ESNs were generated by exposure rather than infection and waning of antibody. Considerable overlap in levels of HLA-DR/CD38 expression was observed between seropositive and ESN HCWs. A study of SARS-CoV-2 ESN HCWs in Swadling et al. (2022) found a significant correlation between the magnitude of the T-cell response and transcript levels of *IFI17*, an IFN-inducible marker of early infection (7). Swadling et al. concluded that transient infection had occurred, but was aborted by early T-cell responses (7). However, ESNs with elevated *IFI17* transcripts only represented 10% of ESNs, and mean *IFI17* expression was lower in ESNs than seropositive HCWs (7). ESNs may therefore represent not one discrete group, but a spectrum of immune engagement, from subclinical exposure to transient infection.

Ogbe et al. (2020) identified SARS-CoV-2-specific T-cell responses in exposed HCWs: three of 10 generated IFN*γ* to S, M and NP, whilst all generated cellular responses to M and NP by proliferation assay, indicating the potentially higher sensitivity of this assay (19). T-cell responses in ESNs were of greater magnitude than unexposed controls for CD4^+^ but not CD8^+^ cells, although both CD4^+^ and CD8^+^ cells targeted a greater number of antigens in ESNs compared to controls. The presence of T-cells targeting multiple antigens in ESNs is supported by da Silva Antunes et al. (2021), where cellular immunity targeting S as well as the rest of the proteome was higher in ESNs compared to controls (28).

Kubitschke et al. (2007) identified T-cell immunity in seronegative HCWs exposed to HCV-contaminated needles (29). CD4^+^ responses occurred in four of 10 individuals within eight weeks of injury but were absent after 2.5 years. Heller et al. (2013) assayed cellular immunity in HCWs exposed to HCV *via* needlestick, cut, or mucosal exposure, and identified HCV-specific proliferation and IFN*γ* production in 48% (n=30) and 42% (n=26) of individuals, respectively (30). Three-quarters of responses were directed towards NSPs. Responses peaked four to six weeks after exposure, unlike canonical HCV infection where immunity generally peaks at seven to 14 weeks (31). The authors suggested this may reflect boosting of pre-existing cellular memory. However, responses were transient and returned to baseline within months, unlike canonical long-lived memory responses (32). The dynamics of cellular immunity in HCV ESNs appears distinct from seropositive infection in its more rapid induction and reduced longevity.

Finally, Clerici et al. (1994) studied eight ESN HCWs following HIV+ needlestick injury. Four to eight weeks after injury, production of interleukin (IL)-2 by T-cells specific for the *env* glycoprotein was observed in six of eight ESNs, compared to only one of nine unexposed controls (33). However, two ESNs seroconverted at six and 19 months after sampling, reflecting early-stage infection rather than exposure.

## Duration and dose

Viruses that have been well-studied for decades provide valuable information on the roles of exposure frequency and response durability in ESNs. Thurairajah et al. (2011) studied seronegative injection drug users exposed to HCV with differing injection behaviours (non-injectors in rehabilitation, infrequent injectors, and continuing injectors) (34). Continuing injectors had stronger and more numerous T-cell responses to HCV compared to non-injectors and healthy controls. Furthermore, individuals who had last injected over 12 months ago had a lower proportion of positive responses than those who had injected in the last six months. These data indicate that ongoing exposure to virus is one factor in the maintenance of T-cell responses in HCV ESNs.

Animal studies also provide insight into the role of antigen exposure for HCV. Shata et al. (2003) demonstrated that two chimpanzees exposed at six month intervals to increasing doses of HCV generated transient T-cell responses (35). 12 months later, both chimpanzees were exposed to a tenfold greater dose of the virus and became infected. The chimpanzee with consistently stronger T-cell responses cleared infection whilst the other developed chronic disease. Interpretation of this result is limited by the small cohort size. Furthermore, macaques exposed to infectious doses of simian immunodeficiency virus seroconverted but generated weak cellular responses, whilst those exposed to sub-infectious doses generated cellular responses only (36). These findings suggest that dose may factor into which arm of adaptive immunity dominates upon viral exposure. Similar challenge studies in primates or humans exposed to differing doses of SARS-CoV-2 would be necessary to make conclusions about the role of dose in SARS-CoV-2 ESNs.

T-cell responses in seronegative household contacts exposed to SARS-CoV-2 suggest that prolonged exposure may not be essential for cellular immunity (7, 19, 28). The durability of these responses is unknown due to the short timescale since virus emergence as well as the confounding influence of vaccination against SARS-CoV-2. Future studies on SARS-CoV-2 ESNs would benefit from sampling high-risk seronegative individuals, but although NP-targeting immunoassays could be used, these studies will be hamstrung by vaccination.

## Target antigens

Determining which antigens are targeted in SARS-CoV-2 ESNs provides insight into mechanisms of response. T-cells targeting the replication-transcription complex (RTC) of SARS-CoV-2 were described by Swadling et al. (2022) in ESNs (7). The RTC is comprised of the RNA polymerase NSP12, a co-factor NSP7, and the helicase NSP13 (37). Its expression early in the SARS-CoV-2 replication cycle makes the RTC a target for rapidly-induced T-cell responses (7). The authors identified fivefold-higher RTC-specific T-cell responses in ESNs compared to unexposed controls. Furthermore, cellular immunity in ESNs preferentially targeted the RTC over structural proteins compared to seropositive individuals. However, the authors did not assay cellular responses to other NSPs. In a study of six ESN sexual partners of HSV-2-infected individuals by Posavad et al. (2010), T cell responses in ESNs were skewed towards peptides expressed early in the virus replication cycle, whereas HSV-2 seropositive individuals more frequently generated responses to structural proteins present in virions (8). The authors speculated that this skew in ESNs reflected early T-cell engagement with infected cells before the production of infectious virions. Together, these data support a model whereby rapid T-cell responses targeting early translated NSPs may prevent infection from gaining a foothold.

## Cytokine profile

In a cohort of 52 household contacts of SARS-CoV-2-infected individuals, Kundu et al. (2022) identified higher frequencies of IL-2-, but not IFN*γ*-, secreting T-cells in ESNs compared to individuals that later became infected (38). A similar study of household contacts from Brand et al. (2021) reported no T-cell recognition of SARS-CoV-2 epitopes in seronegative individuals – this was measured by a novel IFN*γ* assay, which may lack sensitivity for low magnitude responses in ESNs (39). Assays that measure IFN*γ* production alone may underestimate the prevalence of cellular immune responses in ESNs, highlighting the need for multiple sensitive immunophenotyping methods, such as flow cytometric or proliferation assays, to accurately quantify responses.

T_H_1-focussed cytokine production has been described in HBV ESNs (18). Sexual partners of infected individuals generated proliferative T-cell and IFN*γ* responses to HBV peptides. No ESNs generated TNFα or IL-10 responses, unlike seropositive individuals (40). Finally, IFN*γ* secretion was described in seronegative individuals exposed to Ebola virus (EBOV) (41). A study of EBOV close contacts (n=42) from Thom et al. (2021) identified two ESNs. However, responses in these ESNs were not present in further samples, potentially reflecting experimental artefacts. This further highlights the need for sensitive immunophenotyping assays to examine the true prevalence of ESNs.

## Proposed mechanism

To prevent infection before seroconversion, a rapid cellular response appears critical. Chandran et al. (2021) assayed weekly nasopharyngeal swabs and blood samples from HCWs, and demonstrated that SARS-CoV-2 specific T-cell proliferation can occur before PCR positivity (42). These rapid responses may originate from pre-existing, cross-reactive T-cells specific for human coronaviruses (HCoVs). Cross-recognition of SARS-CoV-2 by HCoV-specific T-cells has been widely described (43–50), and T-cells from COVID-19 convalescents preferentially target conserved epitopes over SARS-CoV-2-specific epitopes (49). HCWs display higher levels of HCoV-specific T-cells than community controls (28), which may contribute to the abundance of ESNs amongst HCWs. The activation of cross-reactive T-cells by related viruses has been termed ‘heterologous immunity’ (51). This is distinct from autologous viral infection in that neutralising antibody responses to the heterologous virus may be suboptimal, allowing cellular memory to dominate.

The RTC is highly conserved between SARS-CoV-2 and HCoVs (7). Tetramer staining of T-cells with an HCoV-HKU1 homologue of the RTC component NSP7 showed strong responses in SARS-CoV-2 ESNs. Swadling et al. (2022) suggested that prior exposure to HCoV-HKU1 generates cross-reactive T-cells specific for NSP7, enabling rapid abortion of SARS-CoV-2 infection (7). A study of camel workers in Saudi Arabia identified both CD4^+^ and CD8^+^ responses to Middle-East Respiratory Syndrome coronavirus in four highly-exposed seronegative individuals, suggesting that the ESN phenomenon may be common to other human-infective coronaviruses (52).

It is unclear whether cross-reactive T-cells contribute to ESN immunity in HCV, HIV, HBV or HSV. Cross-reactivity between HCV and influenza A has been described, with HCV-seronegative individuals generating T-cell responses to a cross-reactive HCV epitope (53). However, human viruses with homology to HIV, HBV or HSV have not been described and are thus unlikely to be the driver of the ESN phenomenon for these viruses.

## Correlates of protection from infection

Key to understanding correlates of protection against SARS-CoV-2 infection is deciphering the role of cellular versus humoral immunity. Seropositivity may not always be the most appropriate marker if cellular immunity is protective. This is particularly relevant for assessing vaccine-induced protection against disease where neutralizing antibody titres are a common endpoint, and particularly for SARS-CoV-2 where an arms race between booster vaccination and waning antibody titres has begun.

In a model whereby cellular immunity in ESNs is protective, one would reasonably expect that the magnitude of cellular response in ESNs would be greater than in seropositive individuals, to compensate for the lack of humoral immunity. Cellular immunity is able to clear SARS-CoV-2 infection in isolation; patients with X-linked agammaglobulinemia who cannot produce antibodies eventually clear SARS-CoV-2 infection, and mount higher magnitude CD8^+^ T-cell responses to SARS-CoV-2 compared to immunocompetent individuals (54). However, in Wang et al. (2021) the magnitude of the SARS-CoV-2-specific CD4^+^ T-cell response was twice as high in infected individuals compared to ESNs. This casts doubt on their role in protection against infection.

In influenza virus infection, cytotoxic T-cells target conserved non-structural proteins while antibodies target the divergent neuraminidase and hemagglutinin proteins and are thus strain-specific. In 1983, McMichael and colleagues demonstrated that individuals with cross-reactive T-cells targeting influenza A were able to clear infection in the absence of subtype-specific antibody (55). Later studies showed cross-reactive CD4+ and CD8+ T-cells are associated with milder disease in individuals lacking cross-reactive antibody (56, 57). Animal challenge models have shed light on whether cellular immunity following vaccination can confer protection against influenza. Vaccination of mice with a virus-like particle vaccine against influenza A virus promoted cross-reactive CD8+ T-cell-mediated protection against later challenge with a heterosubtypic strain, supporting the idea that cellular immunity in the absence of subtype-specific antibody can confer protection against infection (58). The applicability of cross-reactive T cell responses in influenza virus models to other virus families is unclear. However, the observations in these studies strongly support cellular immunity being considered in estimates of correlates of protection for viral infection.

## Discussion

**Figure 1:**
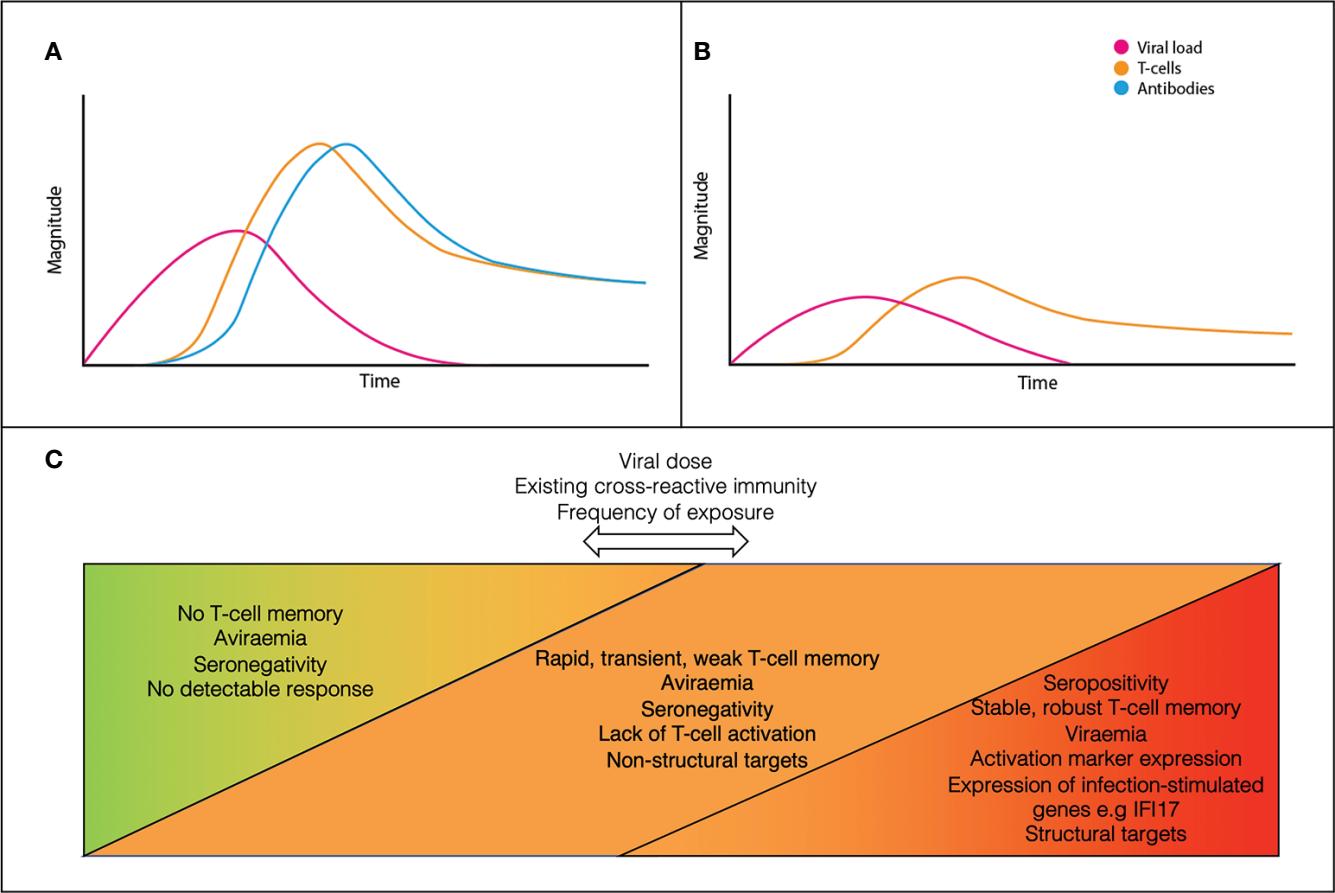
A conceptual model for the dynamics of canonical acute infection **(A)** vs ESN infection **(B)**. ESNs (orange) represent points along a spectrum of immune engagement, influenced by viral dose, immune cross-reactivity, and frequency of exposure **(C)**.

A model for the dynamics of adaptive immunity in infection versus exposure is shown in **Figure 1**. In canonical infection (**Figure 1A**), T-cells and antibodies reduce viral load and contribute to disease resolution. In ESNs (**Figure 1B**), T-cells proliferate alone and at lower levels than canonical infection. Viral load never reaches detectable levels and clinical disease does not occur. This may result from early proliferation of cross-reactive memory T-cells. Rather than being a discrete group, ESNs likely represent points along a spectrum of immune engagement, influenced by viral and host factors (**Figure 1C**). Discrepancies across studies likely reflect individuals at different points along this spectrum, dependent upon viral dose, existing cross-reactive immunity, and frequency of exposure.

**Table 1** displays a summary of findings for SARS-CoV-2 and other viruses. However, significant gaps in our understanding of this phenomenon remain. Specific areas that would provide further clarification include:

1. **Repeated exposure and response durability**. Frequent exposure appears critical in the durability of HCV-specific cellular responses. SARS-CoV-2 challenge studies in primates and humans would clarify the role of dose and exposure frequency in ESNs, durability of responses, and the extent to which cellular immune responses correlate with protection against infection.
2. **Interaction with innate components**. Although not covered here, cross-reactive T-cells likely act in coordination with innate immunity to prevent infection. Natural killer cells have been demonstrated to mediate resistance to HCV infection (59–61), and polymorphisms in immune mediator genes such as *IL28B* likely contribute to disease susceptibility (62). Future research into correlates of protection for SARS-CoV-2 should examine both innate and cellular components in seronegative infection, for example with the use of flow cytometric assays that enable precise dissection of immune components.
3. **Other T-cell subsets.** Many of the studies outlined in this review use whole blood samples representing circulating immunity. It is critical for future research to consider mucosal and tissue-resident cells to generate a complete picture wherever possible (63). This would require additional sampling such as nasopharyngeal swabs or respiratory samples.

Our understanding of the ESN phenomenon remains in its infancy yet offers opportunities for development. The remarkable heterogeneity in outcome following SARS-CoV-2 exposure makes understanding infection susceptibility crucial for prevention and treatment. Significant insight can be gained into correlates of protection against SARS-CoV-2 by further investigating this phenomenon and gaining a deeper understanding of the role of cellular immunity in protection against infection.

**Table 1:** Summary of ESN findings for SARS-CoV-2 and other viruses.

	SARS-CoV-2	Other
**Canonical immune response**	Both humoral and cellular immunity weeks after infection. Resolution usually within weeks with a small percentage experiencing severe or fatal outcomes.	HCV: Adaptive immunity months after infection. Cellular immunity with some contribution from neutralising antibodies.
**Exposure duration and dose**	Prolonged exposure not essential. Duration of exposure required is unclear.	HCV: Ongoing or recent exposure important for response maintenance. Potential role for low doses of virus. Responses peak earlier than in seropositive infection.
**Durability**	Unknown	HCV: Limited, with waning of responses observed within one year
**Target antigens**	Early translated antigens More non-structural targets than seropositive individuals	HCV: Early life cycle peptides
**Cytokine profile**	IL-2, IFN*γ* in some cases but not always	HBV: IFN*γ* production EBOV: IFN*γ* production
**Potential source of response**	Cross-reactivity with HCoVs	MERS: Potential cross-reactivity with HCoVs

## Author contributions

CJ conceived the idea and wrote the manuscript. JR assisted with finalising the manuscript. LT, PG and PK proofread the manuscript. All authors contributed to the article and approved the submitted version.
